# Optimization of Polyphenol Extraction from *Allium ampeloprasum* var. *porrum* through Response Surface Methodology

**DOI:** 10.3390/foods7100162

**Published:** 2018-10-02

**Authors:** Irini F. Strati, George Kostomitsopoulos, Fotios Lytras, Panagiotis Zoumpoulakis, Charalampos Proestos, Vassilia J. Sinanoglou

**Affiliations:** 1 Laboratory of Chemistry, Analysis & Design of Food Processes, Department of Food Science and Technology, University of West Attica, Ag. Spyridonos, 12243 Egaleo, Greece; irinistrati@yahoo.gr (I.F.S.); gk1599@hotmail.com (G.K.); h2ofwths@gmail.com (F.L.); 2 Institute of Biology, Medicinal Chemistry & Biotechnology, National Hellenic Research Foundation, 48, Vas. Constantinou Ave., 11635 Athens, Greece; pzoump@eie.gr; 3 Laboratory of Food Chemistry, Department of Chemistry, National and Kapodistrian University of Athens, 15784 Athens, Greece; harpro@chem.uoa.gr

**Keywords:** *Allium ampeloprasum*, leek, extraction, polyphenols, optimization, Box-Behnken design, antioxidant activity

## Abstract

*Allium ampeloprasum* var. *porrum* has been recognized as a rich source of secondary metabolites, including phenolic acids, flavonoids and flavonoid polymers (proanthocyanidins or condensed tannins), with related health benefits. Both parts of *Allium ampeloprasum* var. *porrum* (white bulb and pseudostem) are traditionally consumed either as a vegetable or as a condiment in many Mediterranean countries. The aim of the present study was to optimize the extraction conditions of polyphenols from white leek stem and green leek leaf by implementing a Box-Behnken design (BBD). The optimization considered basic factors affecting extraction efficiency, including extraction time, solvent to plant material ratio and solvent mixture composition. Maximum polyphenol yield was achieved at an extraction time of 80 and 100 min for white leek stem and green leek leaf extracts respectively, solvent to plant material ratio of 5:1 (*v/w*) and methanol to water ratio of 40:60 (*v/v*), for both leek extracts. Interestingly, higher total phenolic content was found in green leek leaf extracts compared to white leek stem extracts, due to a possible relationship between polyphenol production and sunlight radiation. High correlation values were also observed between total phenolic content and antioxidant-antiradical activity of optimized leek extracts.

## 1. Introduction

Leek (*Allium ampeloprasum* var. *porrum*) belongs to the *Allium* genus and is one of the most important vegetables cultivated in European countries from the Balkan Peninsula to Ireland and in western Asia (e.g., Middle East) [1,2]. Leek is brought to market year round by combining different production methods and cultivars, either in protected conditions or in the field for summer, autumn and winter harvest. Leek is grown for its cylindrical pseudo stem, which is blanched white from growing underground and is made up of the bases of long leaves [3].

*Allium* species including leek (*Allium ampeloprasum* var. *porrum*), chives (*Allium schoenoprasum* L.), onion (*Allium cepa* L.), garlic (*Allium sativum* L.) and Welsh onion (*Allium fistulosum* L.), are consumed as vegetables, medicinal herbs, and food seasonings. They are considered rich sources of secondary metabolites, including phenolic acids and their derivatives, flavonoids (flavan, flavanone, flavones, flavonol, dihydroflavonol, flavan-3-ol, flavan-4-ol and flavan-3,4-diol) and flavonoid polymers (proanthocyanidins or condensed tannins) and therefore have significant health benefits [4,5,6,7,8]. Previous research studies have demonstrated that kaempferol is the main flavonoid aglycone in leek [9,10]. Modern epidemiological research documents a multitude of health benefits for *Allium* species, including anti-asthma, antiseptic, diuretic [11] and antibacterial [12], antioxidant [13], and antifungal [14] properties, the protection of skin against damage due to pathogenic agents [15], and decreased risk of gastrointestinal diseases [16].

Generally speaking, the extraction process is determined by the nature of plant material and the bioactive compounds to be recovered; both of them should be considered in order to achieve good extraction efficiency. The recovery of polyphenols is considered a difficult task, as phenolic compounds exist as free aglycones, as conjugates with sugars or esters, or as polymers with multiple monomeric units. Moreover, phenolic compounds are not uniformly distributed and may be associated with cell walls, carbohydrates, or proteins. Additionally, most plant materials are natural matrices with high enzyme activity and their stability varies significantly.

Solvent extraction is the most common technique employed to obtain extracts with high phenolic content from a broad range of plant origin matrices. Water, methanol, ethanol and acetone are commonly used as extraction solvents. Methanol is often used due to its high extraction efficiency. Mixtures of methanol and ethanol with water are also widely used for extracting phenolics. Aqueous methanol (50–80%, by vol.) has been used to extract phenolic acids flavonoids and flavonoid polymers [17,18,19]. Increasing the water proportion in the solvent mixture facilitates the extraction of polar compounds such as glycosides. Conversely, certain groups of flavonoids, such as flavones and flavanols, due to the complexity of heterosidic combinations, are not generally characterized as intact compounds but by the form of their aglycones [20].

The type of solvent, the extraction time and temperature, the number of extraction cycles, the solvent to plant material ratio, and the extraction technique are the main factors significantly influencing extraction efficiency. Data on the optimization of the extraction of polyphenols from leek (*Allium ampeloprasum* var. *porrum*) are very scarce, and on this basis, this study attempted to optimize the extraction process, for the efficient recovery of polyphenols from white leek stems and green leek leaves. The optimization was based on a Box–Behnken experimental design and the extracts obtained under optimal conditions were further assessed for their antioxidant and antiradical activity.

## 2. Materials and Methods

### 2.1. Reagents and Standards

All solvents and reagents of analytical grade were purchased from Merck KGaA (Darmstadt, Germany), Carlo Erba Reagents (Val de Reuil, France), Tokyo Chemical Industry Co. LTD (Tokyo, Japan), Sigma-Aldrich (Saint Quentin Fallavier, France) and Sigma Chemical (St. Louis, MO, USA).

### 2.2. Plant Material Preparation

Leeks (*Allium ampeloprasum* var. *porrum)*, characterized by a long white stem of uniform length and short green leaves and cultivated under open field conditions were used as the plant material in this study. Fifty leek plants were collected from a local producer in the region of Evvoia (Greece). Immediately after harvest, the leeks were transported to the laboratory; they were washed, the roots and decayed leaves were removed, and the remainder was divided into white stem and green leaf sections. The sections were then chopped into 1 cm^2^ pieces and stored at 4 °C to await further analysis. Prior to extraction, the pieces of both sections of plant material were blended in a domestic food processor. All analysis was performed within 10 days. According to Bernaert et al. [3], the maximum refrigerated storage time in household practices was thirteen days.

### 2.3. Extraction Procedure

The factorial designed experiments (as described in Section 2.7) were conducted with a solvent mixture (methanol:water) composition varying from 50:50 to 90:10 (*v/v*), solvent to plant material ratio varying from 3:1 to 7:1 (*v/w*), and an extraction time between 30 and 90 min. The selection of values for each factor was based on preliminary experimentation and literature data. Approximately 5–6 g of white leek stem and green leek leaf pulp was placed in 50-mL Falcon centrifuge tubes with the appropriate volume of solvent mixture (methanol:water) composition, as defined by the experimental design. Extractions were performed in an orbital and linear motion shaker “Rotaterm” (J.P. Selecta, Cod: 3000435) at 175 rpm, at room temperature (22 ± 2 °C) for predetermined time periods. Upon completion of the extraction, the extracts were filtered and the extract volume was recorded prior to subsequent determinations. 

### 2.4. Determination of Total Phenolic Content (TPC)

The total phenolic content of each sample was determined applying a micro method of Folin–Ciocalteu’s colorimetric assay, as modified by Andreou et al. [21]. Absorbance was measured at room temperature at 750 nm with a Vis spectrophotometer (Spectro 23, Digital Spectrophotometer, Labomed, Inc., Los Angeles, CA, USA). The total phenolic content was expressed as mg gallic acid equivalents (GAE) per 100 g wet weight (ww), using a standard curve with 25–2600 mg/L gallic acid (*y* = 0.0005*x* + 0.0786, *R*^2^ = 0.9989).

### 2.5. Scavenging Activity on 2,2’-Azino-bis-(3-ethylbenzothiazoline-6-sulfonic acid) Radical (ABTS^+^)

The antiradical activity of green leek leaf and white leek stem extracts was determined according to the method described by Lantzouraki et al. [22]. Absorbance was measured at 734 nm with a Vis spectrophotometer (Spectro 23, Digital Spectrophotometer, Labomed, Inc.). A standard curve with a concentration range of 0.20 to 1.50 mM Trolox (*y* = 0.2876*x* − 0.002, *R*^2^ = 0.9995) was used. The antiradical activity of the samples was expressed as mg Trolox equivalents (TE) per 100 g wet weight (ww).

### 2.6. Ferric Reducing/Antioxidant Power Assay (FRAP)

The ferric reducing antioxidant power assay (FRAP), based on the reduction of a ferric-2,4,6-tripyridyl-s-triazine complex to the ferrous form, was performed according to the method described by Lantzouraki et al. [23]. A standard curve (*y* = 0.0030*x* + 0.0065, *R*^2^ = 0.999) using various concentrations (600–2000 μM) of FeSO_4_·7H_2_O stock solutions was used. Results were expressed as mg FeSO_4_·7H_2_O per 100 g wet weight (ww).

### 2.7. Experimental Design and Statistical Analysis

Experiments were conducted in triplicate and the data including the partial correlations were analyzed by STATISTICA software (Statsoft Inc., 2004, Tulsa, OK, USA). Analysis of variance (ANOVA) and Duncan’s multiple range tests were used to determine the significant difference in total phenolic content, at a 95% confidence level (*p* < 0.05).

The extraction parameters, i.e., extraction time (*X*_1_), solvent to plant material ratio (*X*_2_) and solvent mixture (methanol:water) ratio (*X*_3_), were optimized using the Box-Behnken experimental design by means of the software STATISTICA (Statsoft Inc., 2004). The experimental design involved three process variables each at three equidistant levels (−1, 0, +1) and the response variable was the total phenolic content (*Y*). The levels of the three variables were chosen according to preliminary experiments. In total, 15 combinations of process variables were applied. The combination of variables at the center of the level was run in triplicate. The experimental design determined the effect of the three main variables (*X*_1_, *X*_2_ and *X*_3_) and their interactions on the response variable. The effect of each variable and their interactions on total phenolic content was evaluated by using ANOVA technique. In order to describe relationships between response (*Y*) and the experimental variables (*X*_1_, *X*_2_, *X*_3_), a regression model containing 10 coefficients, including linear and quadratic effect on factors and linear effect on interactions, was described by the following equation:

$$Y=\beta_{o}+\sum_{i=1}^{3}\beta_{i}X_{i}+\sum_{i=1}^{3}\beta_{ii}X_{i}^{2}+\sum_{i=1}^{2}\sum_{j=i+1}^{3}\beta_{ij}X_{i}X_{j},$$

where *β_ο_* is the constant coefficient (model intercept), *β_i_* is the linear coefficient of the main factors, *β_ii_* is the quadratic coefficient for the main factors, and *β_ij_* is the second order interaction coefficient. The 3D response graphs and profile for the predicted values and desirability level for factors were plotted using STATISTICA software.

## 3. Results

A Box-Behnken experimental design applied for 3 variables was used to optimize the extraction of polyphenols from green leek leaf and white leek stem extracts and to determine the combined effect of the extraction time (*X*_1_), the solvent to plant material ratio (*X*_2_) and the solvent mixture composition (*X*_3_) on the total phenolic content (TPC) (*Y)*. Table 1 presents the levels of the independent process variables (*X*_1_, *X*_2_ and *X*_3_) in their coded and actual form, according to the experimental design and the observed and predicted values of the response *Y* (mg GAE/100 g wet weight) for all experiments. The experiments were randomized in order to maximize the effects of unexplained variability in the observed responses due to extraneous factors.

The correlation between total phenolic content (*Y*) and the three processing variables (*X*_1_, *X*_2_ and *X*_3_) were described by two second-order polynomial equations, for both sections of leek, as shown in Table 2.

Both equations found that TPC can adequately predict the total phenolic content at different levels of three process variables influencing the extraction, as the lack of fit was statistically significant (*p* < 0.05) and close to the observed ones (*R*^2^ = 0.98 and 0.92), for green leek leaf and white leek stem extracts, respectively.

Taking into consideration the analysis of variance (ANOVA) (Table 3) and on the basis of the *F*-test, for green leek leaf extracts, the extraction time (*X*_1_) and solvent to plant material ratio (*X*_2_) had significant (*p* < 0.05) quadratic effect on total phenolic content, whereas only the interactions between extraction time (*X*_1_) and solvent to plant material ratio (*X*_2_) and between extraction time (*X*_1_) and methanol:water ratio (*X*_3_), had a significant (*p* < 0.05) effect on total phenolic content. Additionally, for white leek stem extracts, both solvent to plant material ratio (*X*_2_) and methanol:water ratio (*X*_3_) had significant (*p* < 0.05) linear and quadratic effects on total phenolic content, whereas the extraction time (*X*_1_) had only quadratic effect on total phenolic content. Finally, the interaction between extraction time (*X*_1_) and solvent to plant material ratio (*X*_2_) and between solvent to plant material ratio (*X*_2_) and methanol:water ratio (*X*_3_), had a significant (*p* < 0.05) effect on total phenolic content.

The theoretical calculation of the optimum conditions for extraction of total phenols was evaluated by the non-linear optimization algorithm and a maximum total phenolic content of 22.114 and 17.620 mg GAE/100 g ww was achieved, for green leek leaf and white leek stem extracts, respectively. These maximum values were computed under optimum conditions; more specifically, at 100 and 80 min extraction time, for green leek leaf and white leek stem extracts respectively, and at solvent to plant material ratio 5:1 (*v/w*) and methanol:water composition 40:60 (*v/v*) for both extracts.

**Figure 1:**
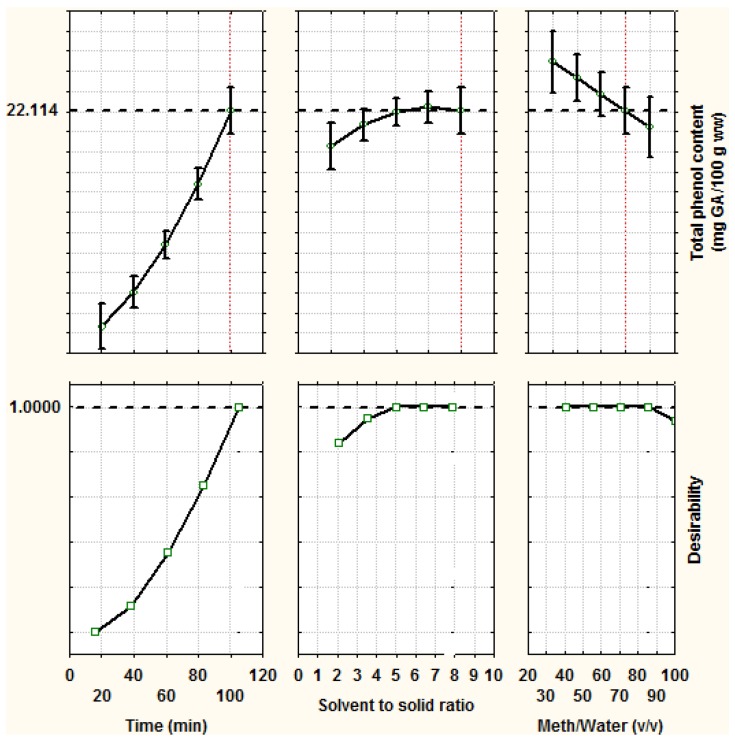
Predicted total phenol content and desirability level for different variables of optimum polyphenol extraction from green leek leaf extracts.

**Figure 2:**
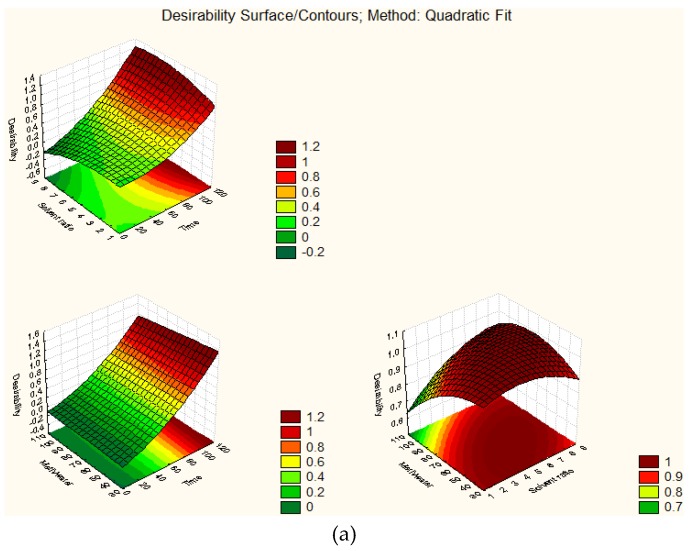

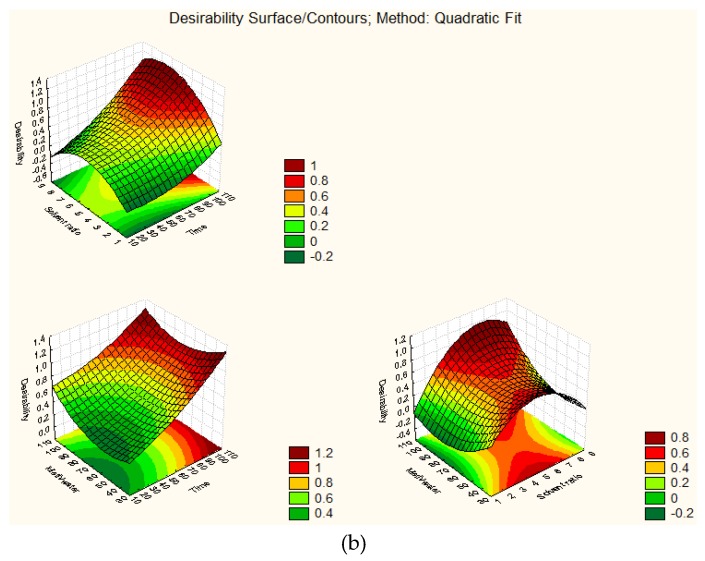
Desirability surface/contour plots (**a**) green leek leaf extracts; (**b**) white leek stem extracts.

The desirability levels for the three extraction variables for optimum polyphenol extraction are presented either as profiles or desirability surface/contour plots in Figure 1 and Figure 2, indicating that the maximum desirability of 1.0 (in a scale of 0–1) can be achieved with the aforementioned optimum conditions for both extractions. The total phenol content and desirability level reduced considerably when the extraction time was less than 100 and 80 min, for green leek leaf and white leek stem extracts, respectively. Additionally, for both extracts, the desirability level reached the maximum value of 1, between solvent to plant material ratio of 5:1 and 7:1 (*v/w*), and methanol:water composition of 40:60 and 90:10 (*v/v*); therefore, it is recommended to use the minimum solvent to plant material ratio and methanol:water composition, for reasons of cost, disposal and ease of separation from final extract.

The optimized extracts of green leek leaf and white leek stem were further analyzed in order to determine their antiradical and antioxidant activity, by ABTS and FRAP assays. Results for ABTS and FRAP assays, expressed as mg of Trolox equivalents (TE) and Fe(II) respectively, per 100 g of leek wet weight (ww), ranged from 31.03 to 36.56 mg TE/100 g ww and from 5.39 to 7.25 mg Fe(II)/100 g ww. Furthermore, correlation analysis was performed and a strong positive correlation was noticed between the TPC and the antiradical activity (0.74, *p* < 0.05), between the TPC and the antioxidant activity (0.86, *p* < 0.05), as well as between antiradical and antioxidant activity (0.78, *p* < 0.05).

## 4. Discussion

The experimental values of total phenolic content obtained with different combinations of independent variables, varied from 5.579 to 21.456 and from 5.906 to 12.863 mg GAE/100 g wet weight (ww), for green leek leaf and white leek stem extracts, respectively. The above values are comparable to those reported for leeks extracted with 80% ethanol and ranging from 210.67 ±16.63 mg/kg to 254.80 ±10.09 mg/kg [24]. Another study determined the content of total phenols in the ultrasonic extracts of leek *Allium porrum* L. treated with ethanol and total phenol content (TPC) was found to be 45.39 and 69.46 mg gallic acid equivalent (GAE)/g dry extract, for leaf and stem extract, respectively [25]. Our results are lower than those of 30 leek cultivars reported by other researchers [26], with values ranging from 74.87 to 196.84 mg GAE 100 g^−1^ fresh weight (fw) in the white stem and from 77.13 to 213.47 mg GAE 100 g^−1^ fw in the green leaves. In agreement with the above findings, previous studies [13,27] reported average total phenolic content of wild leek as 5.77 mg GAE/g extract, whereas the respective values were found to be considerably lower for *A. porrum* (0.369 mg GAE/g extract). The U.S. Department of Agriculture [28] reported a total phenolic content of 47 mg GAE 100 g^−1^ fw in the bulb and lower leaves of leek.

The comparison of results of several research studies is not always appropriate to estimate extraction efficiency due to variations in plant characteristics, such as plant cultivars, growing seasons and agricultural practices. Additionally, the type of solvent and the extraction technique can influence the total phenol content as well as the variation of moisture content in the original plant, which can affect the expression of results.

Finally, in our study green leek leaf extracts contained higher amounts of total phenolics in comparison with white leek stem extracts, identifying a possible correlation between increased polyphenol production and exposure to sunlight radiation, as previously reported in St. John’s wort [29] and barley [30]. Moreover, the high correlation values found among TPC, FRAP and ABTS assays indicate the significant contribution of the phenolic compounds contained in the optimized leek extracts in their antiradical and antioxidant activity. According to Bernaert et al. [31] and Soininen et al. [32], the main flavonoids identified in *Allium* species were kaempferol and quercetin derivatives. Among the methods for determining the antioxidant capacity in vitro, DPPH, FRAP and ORAC assays are the most widely used. For these assays, different data may be obtained, due to different activity patterns of the sample antioxidants in each method. Therefore, several methods and standards should be used and their results compared in order to confirm the antioxidant capacity of a complex sample. FRAP is the only assay that directly measures antioxidants in a sample and provides information about the ability of a compound to reduce ferric complex ion, whereas DPPH, ORAC and ABTS assays are indirect, because they measure the inhibition of reactive species (free radicals) generated in the reaction mixture, and their results also depend strongly on the type of reactive species used. In a previous study [26], extracts of the white shaft and green leaves of 30 leek cultivars were investigated for their antioxidant properties and the white leek shaft had an antioxidant activity of 57 μmol TE g^−1^ dry weight (dw) (ORAC), 9 μmol Fe_2_SO_4_ g^−1^ dw (FRAP) and 6 μmol TE g^−1^ dw (DPPH), whereas the green leaves had an antioxidant activity of 101 μmol TE g^−1^ dw (ORAC), 27 μmol Fe_2_SO_4_ g^−1^ dw (FRAP) and 9 μmol TE g^−1^ dw (DPPH). Additionally, based on the results of previous study [13], wild populations of *Allium ampeloprasum* L. had low antioxidant activity measured by DPPH and inhibition of β-carotene bleaching, and a moderate-high antioxidant activity measured by TBARS and reducing power methods.

## 5. Conclusions

Conclusively, response surface methodology was effectively applied to optimize the phenolics extraction from white stems and green leaves of *Allium ampeloprasum*. Based on the regression model computed, the most significant parameters affecting phenolics recovery were the extraction time, the solvent to plant material ratio (*v/w*) and the solvent mixture composition (methanol:water, *v/v*). Moreover, the optimum conditions attained in the present work could be implemented to similar plant materials.

## Figures and Tables

**Table 1:** Independent variables, their coded (actual) levels and the corresponding observed and predicted responses.

Run No. ^1^	*X*_1_	*X*_2_	*X*_3_	Green Leaf		White Stem	
				*Y*-Observed	*Y*-Predicted	*Y*-Observed	*Y*-Predicted
1	−1 (30)	−1 (3)	0 (70)	6.886	6.893	5.906	5.535
2	+1 (90)	−1 (3)	0 (70)	17.173	17.538	9.974	9.771
3	−1 (30)	+1 (7)	0 (70)	4.311	3.946	6.073	6.275
4	+1 (90)	+1 (7)	0 (70)	18.762	18.754	12.606	12.977
5	−1 (30)	0 (5)	−1 (50)	6.456	6.086	6.688	7.339
6	+1 (90)	0 (5)	−1 (50)	21.456	20.729	12.863	13.344
7	−1 (30)	0 (5)	+1 (90)	5.579	9.909	8.911	8.590
8	+1 (90)	0 (5)	+1 (90)	16.867	15.403	14.391	10.753
9	0 (60)	−1 (3)	−1 (50)	11.619	11.451	8.261	8.498
10	0 (60)	+1 (7)	−1 (50)	10.186	10.505	9.523	7.727
11	0 (60)	−1 (3)	+1 (90)	10.825	9.754	6.559	9.855
12	0 (60)	+1 (7)	+1 (90)	9.782	10.751	10.484	9.946
13	0 (60)	0 (5)	0 (70)	10.995	11.417	9.365	9.223
14	0 (60)	0 (5)	0 (70)	11.272	11.417	9.028	9.223
15	0 (60)	0 (5)	0 (70)	11.98468	11.417	9.275	9.223

Values in parenthesis indicate actual levels; ^1^ experiments were performed in random order; *X*_1_: Extraction time (min); *X*_2_: Solvent to plant material ratio (*v/w*); *X*_3_: Solvent mixture composition (methanol: water, *v/v*).

**Table 2:** Polynomial equations and statistical parameters calculated after implementation of a three-factor Box-Behnken experimental design.

Response Variable (Total Phenolic Content, mg GAE/100 g)	2nd Order Polynomial Equations	*R* ^2^	*p*
Leek green leaf	1.53 + 0.08*X*_1_ + 0.00*X*_1_^2^ + 0.56*X*_2_ − 0.20*X*_2_^2^ + 0.05*X*_3_ − 0.00*X*_3_^2^ + 0.02*X*_1_*X*_2_ − 0.00*X*_1_*X*_3_ + 0.00*X*_2_*X*_3_	0.98	0.00
Leek white stem	10.92 − 0.02*X*_1_ + 0.00*X*_1_^2^ + 2.00*X*_2_^2^ − 0.32*X*_2_^2^ − 0.30*X*_3_ + 0.00*X*_3_^2^ + 0.01*X*_1_*X*_2_ − 0.00*X*_1_*X*_3_ + 0.02*X*_2_*X*_3_	0.92	0.00

**Table 3:** Analysis of variance (ANOVA) for the total phenol content (*Y*) from leek extracts as a function of extraction time (*X*_1_), solvent to plant material ratio (*X*_2_), solvent mixture (methanol:water) composition (*X*_3_) and their interactions.

Source	Sum of Squares		Degrees of Freedom		Mean Sum of Squares		*F* Value		*p* Value	
	G ^1^	W ^2^	G ^1^	W ^2^	G ^1^	W ^2^	G ^1^	W ^2^	G ^1^	W ^2^
Model	1447.13	319.68	9	9	160.79	35.52	120.44	30.38	0.00	0.00
Intercept	0.10	5.16	1	1	0.10	5.16	0.08	4.41	0.78	0.04
Extraction time (*X*_1_)	2.87	0.23	1	1	2.87	0.23	2.15	0.19	0.15	0.66
Solvent to plant material ratio (*X*_2_)	0.46	6.25	1	1	0.46	6.25	0.35	5.35	0.56	0.02
Methanol:water ratio (*X*_3_)	0.24	9.28	1	1	0.24	9.28	0.18	7.94	0.67	0.01
*X*_1_^2^	20.33	7.77	1	1	20.33	7.77	15.23	6.64	0.00	0.01
*X*_2_^2^	9.54	25.56	1	1	9.54	25.56	7.15	21.86	0.01	0.00
*X*_3_^2^	0.01	8.74	1	1	0.00	8.74	0.00	7.48	0.95	0.01
*X*_1_ × *X*_2_	17.33	6.07	1	1	17.33	6.07	12.98	5.20	0.00	0.03
*X*_1_ × *X*_3_	16.18	1.16	1	1	16.18	1.16	12.12	1.00	0.00	0.32
*X*_2_ × *X*_3_	0.15	7.26	1	1	0.15	7.26	0.11	6.21	0.74	0.02
Residual	69.42	61.96	52	53	1.34	1.17				
Total	1516.56	381.64	61	62						

^1^ Green leek leaf extracts; ^2^ White leek stem extracts.
